# Correlation Between mRNA Expression of Activated Eosinophils and Air Pollutant Exposure in Patients With Asthma

**DOI:** 10.1002/iid3.70065

**Published:** 2024-11-22

**Authors:** Ting‐Yu Lin, Po‐Jui Chang, Chun‐Yu Lo, Hsiao‐Chi Chuang, Chung‐Shu Lee, Chih‐Hao Chang, Chih‐Teng Yu, Meng‐Heng Hsieh, Chien‐Ying Liu, Chih‐Hsi Scott Kuo, Shu‐Min Lin

**Affiliations:** ^1^ Department of Thoracic Medicine Chang Gung Memorial Hospital Taipei Taiwan; ^2^ College of Medicine Chang Gung University Taoyuan Taiwan; ^3^ National Heart and Lung Institute Imperial College London London UK; ^4^ Division of Pulmonary Medicine, Department of Internal Medicine Shuang Ho Hospital, Taipei Medical University New Taipei City Taiwan; ^5^ School of Respiratory Therapy, College of Medicine Taipei Medical University Taipei Taiwan; ^6^ Cell Physiology and Molecular Image Research Center Wan Fang Hospital, Taipei Medical University Taipei Taiwan; ^7^ Graduate Institute of Medical Sciences, College of Medicine Taipei Medical University Taipei Taiwan; ^8^ Department of Pulmonary and Critical Care Medicine New Taipei Municipal Tucheng Hospital New Taipei City Taiwan

**Keywords:** air pollution, asthma, eosinophil, mRNA

## Abstract

**Background:**

Eosinophil activation is associated with asthma. Whether air pollution affects the activation of blood eosinophils in patients with asthma remains unknown. In this study, we investigated the correlation between transcriptional activity in eosinophils and air pollutant exposure in patients receiving different levels of Global Initiative for Asthma (GINA) treatment.

**Methods:**

We evaluated the expression levels of activation‐ and function‐related genes in eosinophils from patients with GINA 4 or 5 (*n* = 20), those with GINA 3 (*n* = 12), and normal individuals (*n* = 7); the eosinophils were activated with interleukin (IL)−5 or IL‐17. A land use regression model was used to estimate air pollutant exposure. The correlations between mRNA expression, lung function, and air pollutant exposure were investigated.

**Results:**

The expression levels of TGFB1, IL7R, and TLR3 were significantly higher for patients with GINA 4 or 5 than for those with GINA 3 or normal individuals. The expression of certain genes, particularly in IL‐17‐activated eosinophils, was correlated with lung function decline in patients with GINA 4 or 5. For patients with GINA 4 or 5, NO_2_ exposure was correlated with upregulated TGFB1 expression in IL‐5‐activated eosinophils. For patients with GINA 3, O_3_ exposure was correlated with upregulated CCR5, IL5RA, IL7R, and TGFB1 expression in IL‐17‐activated eosinophils and upregulated IL7R expression in IL‐5‐activated eosinophils.

**Conclusion:**

Patients with GINA 4 or 5 may exhibit elevated transcriptional activity in eosinophils; this elevation is correlated with lung function decline. Air pollution may affect eosinophil mRNA expression in patients with asthma.

## Graphical abstract

Asthma patients With GINA 4 or 5 exhibit elevated transcriptional activity in eosinophils; this elevation is correlated With lung function decline. Air pollution may affect eosinophil mRNA expression in patients With asthma.

**Abbreviations**
CCR: C‐C motif chemokine receptor
CRLF2: cytokine receptor‐like factor 2
CRTH2: prostaglandin D2 receptor 2
FCER1A: high affinity immunoglobulin epsilon receptor subunit alpha
GINA: global initiative for asthma
ICS: inhaled corticosteroid
IL17RA: interleukin‐17 receptor A
IL4RA: interleukin‐4 receptor α
IL5RA: interleukin‐5 receptor α
IL7R: interleukin‐7 receptor
ITGA4: Integrin alpha‐4
ITGB2: Integrin beta‐2
LABA: long‐acting β agonist
PAL: persistent airflow limitation
PCR: polymerase chain reaction
TGFB1: transforming growth factor β1
TLR: toll‐like receptor

## 1 Introduction

Airway eosinophils participate in airway inflammation and remodeling in patients with asthma [1, 2, 3]. Activated eosinophils undergo piecemeal degranulation to release functional granules or form extracellular DNA traps, leading to airway epithelial damage and mucus plug formation in uncontrolled asthma [4, 5, 6]. Before migration to airway, blood eosinophils are also known as the biomarker of lung function decline and acute exacerbation in patients with asthma [7, 8]. Surface markers (e.g., integrins, cytokines, and chemokine receptors) indicate the activation of blood eosinophils that are recruited to the airway [9, 10, 11]. Evidence suggests that the surface markers of blood eosinophils are correlated with the features of asthma [10]. Allergen‐ or cytokine‐ stimulation mediated priming of blood eosinophils induces a functional response in patients with asthma [12, 13, 14]. Discover the factors contributing to eosinophil activation are important to plan a better preventive strategy of asthma progression.

Particle matter (PM)_10_, PM_2.5_, and gaseous pollutants such as nitrogen dioxide (NO_2_) and ozone (O_3_) are well‐known pollutants that can cause various respiratory diseases [15, 16]. Studies have revealed that exposure to air pollutants is correlated with the development and exacerbation of asthma [17, 18, 19]. However, whether environmental exposure leads to the activation of blood eosinophils in patients with asthma remains unknown.

In the present study, we evaluated the expression levels of activation‐ and function‐related genes in interleukin (IL)‐5‐ or IL‐17‐activated eosinophils derived from patients receiving Global Initiative for Asthma (GINA) 4 or 5 treatment and those receiving GINA 3 treatment. In addition, we investigated the correlations between transcriptional activity in activated eosinophils, air pollutant exposure, and lung function decline to estimate the effects of environmental exposure on the activation of blood eosinophils in patients with asthma. The present study provided a new insight in the immunologic difference in asthma by treatment control levels and how air pollutions correlated it.

## 2 Patients and Methods

### 2.1 Study Design and Population

This study included adult patients with asthma who were regularly monitored at our outpatient clinic for at least 1 year. Asthma was diagnosed on the basis of various respiratory symptoms, such as wheezing, dyspnea, and cough, as well as relevant medical history. For anamnesis, the following conditions were considered: > 12% and > 200 mL improvement in forced expiratory volume in 1 s (FEV1) after bronchodilator use, < 8 mg/mL PC20 in the methacholine test, and > 10% diurnal variation in peak expiratory flow. Eligible patients were nonsmokers and had no respiratory or systemic infection within 2 months before recruitment. We collected data on patients' lung function parameters, blood eosinophil counts, immunoglobulin E (IgE) levels (ImmunoCAP, Phadia, Sweden), and residence in the previous 6 months. Patients who tested positive for IgE antibodies against various allergens (> 0.35 KU/L) were regarded as atopic individuals.

Asthma was managed in accordance with the 2018 GINA guidelines [20]. In general, patients with GINA 4 or 5 were managed with medium to high doses of inhaled corticosteroids (ICSs) combined with long‐acting β agonizts (LABAs), triple inhalers (e.g., ICS + LABA + long‐acting muscarinic antagonist), or biologics. Patients with GINA 3 were managed with low doses of ICS combined with LABA. Individuals who had normal pulmonary function, no respiratory symptoms, and no history of any chronic respiratory disease were included as the control group (normal individuals). All participants provided written informed consent. This study was approved by the Institutional Review Board of Chang Gung Medical Foundation (approval number: 202100988A3C501).

### 2.2 Isolation of Eosinophils From Peripheral Blood

Peripheral blood samples (50 mL) were collected from the participants. The samples were processed through Ficoll–Hypaque density centrifugation (GE Healthcare, Bio‐Sciences AB, Uppsala, Sweden). The lower cell layer, which mainly comprises granulocytes and erythrocytes, was treated using an erythrocyte lysis solution (Bioman Scientific, New Taipei City, Taiwan). Eosinophils were isolated by depleting CD16+ neutrophils; for this, we used an eosinophil isolation kit (Miltenyi Biotec, Auburn, CA, USA). The purity of the collected eosinophil samples was assessed through cytospin. Purified (> 95%) eosinophils were resuspended in Roswell Park Memorial Institute 1640 medium (Gibco Life Technologies, Grand Island, NY, USA) supplemented with 100 U/mL penicillin G, 10 μg/mL streptomycin, 3 μg/mL l‐glutamine, and 20% heat‐inactivated fetal calf serum.

### 2.3 Quantitative Real‐Time Polymerase Chain Reaction

Eosinophils were cultured in 24‐well plates (density, 5 × 10^5^ cells/well) containing 500 μL Roswell Park Memorial Institute 1640 medium. The cells were activated with IL‐5 (25 ng/mL; R&D Systems, Minneapolis, MN, USA), IL‐17α (25 ng/mL; R&D Systems), or phosphate‐buffered saline (control; Sigma‐Aldrich, St. Louis, MO, USA) for 0.5, 1, or 3 h. Next, the cells were harvested for total RNA isolation, which was performed using the TOOLSmart RNA Extractor (Biotools, Taipei, Taiwan). Complementary DNA was synthesized using 1–100 μg of purified RNA. Reverse transcription was performed using the ToolsQuant2 Fast RT Kit (Biotools). For real‐time polymerase chain reaction (PCR), primer sets were manually designed using the Primer‐BLAST tool (National Center for Biotechnology Information). Expression levels of the following genes were analyzed: cytokine receptors—IL5RA, IL17RA, IL4RA, prostaglandin D2 receptor 2 (CRTH2), cytokine receptor‐like factor 2 (CRLF2), and IL7R (proxy: thymic stromal lymphopoietin [TSLP]); adhesion molecules and chemokine receptors—integrin α4 (ITGA4), integrin β2 (ITGB2), C‐C motif chemokine receptor (CCR)3, and CCR5; and various markers—High affinity immunoglobulin epsilon receptor subunit alpha (FCER1A), transforming growth factor β1 (TGFB1), aryl hydrocarbon receptor (AHR), and endosomal Toll‐like receptors (TLRs). Supporting Information S1: Table S1 presents a list of primers used for quantitative real‐time PCR. This analysis was performed using the MyGo Pro real‐time PCR System (Azura Genomics, Raynham, MA, USA) and 2× SuperFast SYBR qPCR Reagent (Biotools). The PCR protocol was as follows: initial denaturation at 95°C for 1 min; 40 cycles of denaturation at 95°C for 5 s, annealing at 60°C for 10 s; extension at 72°C for 15 s; and final extension at 72°C for 10 min. Ct values were derived from the reaction data. Gene expression was normalized against β‐actin expression. Relative gene expression was calculated on the basis of Ct values against the control treatment (phosphate‐buffered saline). Data were calculated using the 2^−∆∆Ct^ method.

### 2.4 Measurement of Air Pollutant Exposure

On the basis of the participants' residence data, we calculated their exposure to PM_10_, PM_2.5_, NO_2_, and O_3_; for this, we used a land use regression model, as described previously [21, 22]. In brief, data on 1‐, 3‐, and 5‐year average levels of air pollutants were obtained from air quality monitoring stations operated by the Taiwan Environmental Protection Administration (https://airtw.epa.gov.tw/). The supervised forward linear regression method was used for constructing the land use regression model. This approach ensures the inclusion of predictors with a plausible direction of effect while maximizing the model's predictive accuracy. Model construction began by incorporating the predictor variable that exhibited the highest adjusted explained variance (R^2^). To compare baseline differences in baseline eosinophil activation (PBS, 0.5 h) and IL‐5 or IL‐17 stimulation (0.5 h) of asthma patients with low or high exposures, medium levels of 1‐year PM_10_ (25.38 μg/m^3^), PM_2.5_ (14.0 μg/m^3^), NO2 (15.12 ppb) and O_3_ (29.08 ppb) exposure of all patients were identified. The 1‐year average exposure level equal to or higher than medium level of exposure was identified as patients with high exposure, otherwise, the patients were identified as with low exposure.

### 2.5 Statistical Analysis

Continuous variables were analyzed using one‐way ANOVA or unpaired Student's *t*‐test, and categorical variables were analyzed using Fisher's exact test. For each time point, between‐group comparisons were performed using the Kruskal–Wallis test, followed by the Dunn–Bonferroni post hoc test. Mann Whitney test was performed for patients with high versus low exposure. The correlations between mRNA expression levels in activated eosinophils (treated with IL‐5 or IL‐17 for 0.5 or 1 h), lung function parameters, and average air pollutant exposure levels were investigated by calculating the corresponding Spearman's rank correlation coefficients. Heatmaps were constructed to visualize the correlations between the average levels of air pollutant exposure and the expression levels of target genes in activated eosinophils. A *p* value of < 0.05 was considered to be significant. Data were analyzed using Prism (version 8; GraphPad Software, La Jolla, CA, USA).

## 3 Results

### 3.1 Study Cohort

This study included 32 patients with asthma (GINA 4 or 5: 20 patients; GINA 3: 12 patients) and 7 normal individuals. Oral corticosteroids and biologics were prescribed to 15% and 25% of all patients with GINA 4 or 5, respectively (Table 1). No significant difference was noted between patients with GINA 4 or 5 and those with GINA 3 in terms of sex, age, age at onset, body mass index, or average air pollutant exposure. The level of IgE exhibited a declining trend in patients with GINA 4 or 5 compared with patients with GINA 3, but the difference was nonsignificant. Lung function was significantly lower in patients with GINA 4 or 5 than in those with GINA 3.

**Table 1:** Characteristics of asthma patients with GINA 4 or 5‐ or GINA 3.

Characteristics	GINA 4 or 5 (*N* = 20)	GINA 3 (*N* = 12)	Normal subjects (*N* = 7)	*p* Value
Age (years) ± SD	53.5 ± 17.5	45.5 ± 18.7	38.1 ± 7.0	0.100
Males, *n* (%)	8 (40.0)	6 (50.0)	2 (28.6)	0.357
BMI (kg/m^2^)	26.3 ± 4.1	28.2 ± 5.2	23.7 ± 2.0	0.098
Age of onset, year ± SD	40.9 ± 18.6	30.2 ± 19.2	—	0.130
IgE level, KU/L	399.0 ± 442.3	943.3 ± 1270	—	0.090
Eosinophils count, cells/μL	110.1 ± 68.8	157.6 ± 146.1	78.6 ± 40.5	0.204
Atopy, *n* (%)	12 (60.0)	8 (66.7)	159 (38.3)	0.706
History of exacerbation in previous year, *n* (%)	3 (15.0)	2 (20.0)	—	0.900
**Lung function**				
FVC, L	2.7 ± 1.1	3.2 ± 0.8	3.4 ± 0.4	0.258
FVC, %	77.0 ± 15.3*, # ^,^ #	93.5 ± 16.4*	106.0 ± 15.8#	0.001
FEV1, L	2.0 ± 0.9	2.4 ± 0.6	2.4 ± 1.1	0.371
FEV1, %	67.9 ± 16.7* ^,^ ¶	83.7 ± 16.1*	104.2 ± 14.6¶	< 0.001
FEV1/FVC, %	71.6 ± 11.3&	75.5 ± 9.6	87.0 ± 3.6&	0.017
Post‐BD FEV1, L	2.2 ± 1.0	2.5 ± 0.6	2.9 ± 0.4	0.214
Post‐BD FEV1, % change	12.7 ± 11.8	6.0 ± 4.8	3.0 ± 2.6	0.058
**Treatment, *N*, (%, Patients may have more than one regimen)**				
Low dose ICS + LABA	0	12 (100%)	**—**	
Medium dose ICS + LABA	8 (40%)	0	**—**	
High dose ICS + LABA	5 (25%)	0	**—**	
Triple inhalers	7 (35%)	0	**—**	
OCS	3 (15%)			
Biologic	5 (25%)	0	**—**	
**Mean exposure to PM**_**10**_, **μg/m^3^**				
1‐year	26.5 ± 3.5	27.1 ± 6.5	26.6 ± 4.6	0.917
3‐year	32.3 ± 3.9	33.7 ± 5.6	32.5 ± 4.1	0.750
5‐year	35.3 ± 4.0	36.9 ± 5.0	35.4 ± 3.9	0.659
**Mean exposure to PM**_**2.5**_, **μg/m^3^**				
1‐year	14.0 ± 2.2	14.3 ± 1.1	15.4 ± 2.5	0.288
3‐year	17.5 ± 2.7	17.2 ± 1.3	18.5 ± 2.7	0.523
5‐year	19.7 ± 2.8	18.9 ± 1.4	20.7 ± 2.6	0.308
**Mean exposure to NO**_**2**_, **ppb**				
1‐year	15.7 ± 4.1	13.2 ± 4.0	16.2 ± 4.3	0.200
3‐year	17.5 ± 4.7	13.7 ± 3.5	17.3 ± 4.3	0.088
5‐year	18.5 ± 4.9	14.6 ± 3.6	18.2 ± 4.4	0.091
**Mean exposure to O**_**3**_, **ppb**				
1‐year	27.6 ± 2.5	29.5 ± 3.2	28.9 ± 1.9	0.167
3‐year	27.3 ± 2.6	29.7 ± 3.3	29.1 ± 2.0	0.101
5‐year	26.8 ± 2.6	29.1 ± 3.3	28.5 ± 2.0	0.116

*Note:* Data are presented in terms of frequency and percentage or mean and standard deviation values, unless otherwise indicated.

Abbreviations: BD, bronchodilator; FEV1, forced expiratory volume in 1 s; FVC, forced vital capacity; GINA, Global Initiative for Asthma; ICS, inhaled corticosteroid; IgE, immunoglobulin E; LABA, long‐acting β agonist; NO_2_, nitrogen dioxide; O_3_, ozone; OCS, oral corticosteroid; PM_10_, particles with an aerodynamic diameter of ≤ 10 μm; PM_2.5_, particles with an aerodynamic diameter of ≤ 2.5 μm.

* *p* < 0.05, GINA 4 or 5 versus GINA 3.

^&^ *p* < 0.05, GINA 4 or 5 versus normal individuals.

^#^ *p* < 0.01, GINA 4 or 5 versus normal individuals.

^¶^ *p* < 0.001, GINA 4 or 5 versus normal individuals.

### 3.2 Transcriptional Activity in Activated Eosinophils From Patients With Asthma and Normal Individuals

Figure 1 depicts the expression levels of cytokine receptors in activated eosinophils. The expression level of IL5RA (Figure 1A) in IL‐5‐activated eosinophils and the levels of IL17RA and CRTH2 (Figure 1B,D) in IL‐5 or IL‐17‐activated eosinophils were significantly higher for patients with asthma than for normal individuals. Notably, the level of mRNA expression was higher for patients with GINA 4 or 5 than for those with GINA 3. Furthermore, the expression level of IL5RA in eosinophils treated with IL‐17 for 0.5 h was higher for patients with GINA 3 than for normal individuals. The expression of IL7R (Figure 1F) in eosinophils treated with IL‐5 or IL‐17 for 0.5 h was significantly higher for patients with GNIA 4 or 5 than for normal individuals.

Figure 1Levels of mRNA expression in eosinophils activated using interleukin‐5 or interleukin‐17 for 0.5, 1, or 3 h. (A) IL5RA. (B) IL17RA. (C) IL4RA. (D) CRTH2. (E) CRLF2. (F) IL7R. Black bars represent patients with GINA 4 or 5, gray bars represent patients with GINA 3, unshaded bars represent normal individuals. Post hoc analysis *p < 0.05 and **p < 0.01. CRLF2, cytokine receptor‐like factor 2; CRTH2, prostaglandin D2 receptor 2; IL17RA, interleukin‐17 receptor α; IL4RA, interleukin‐4 receptor α; IL5RA, interleukin‐5 receptor α; IL7R, interleukin‐7 receptor.

**Figure:**
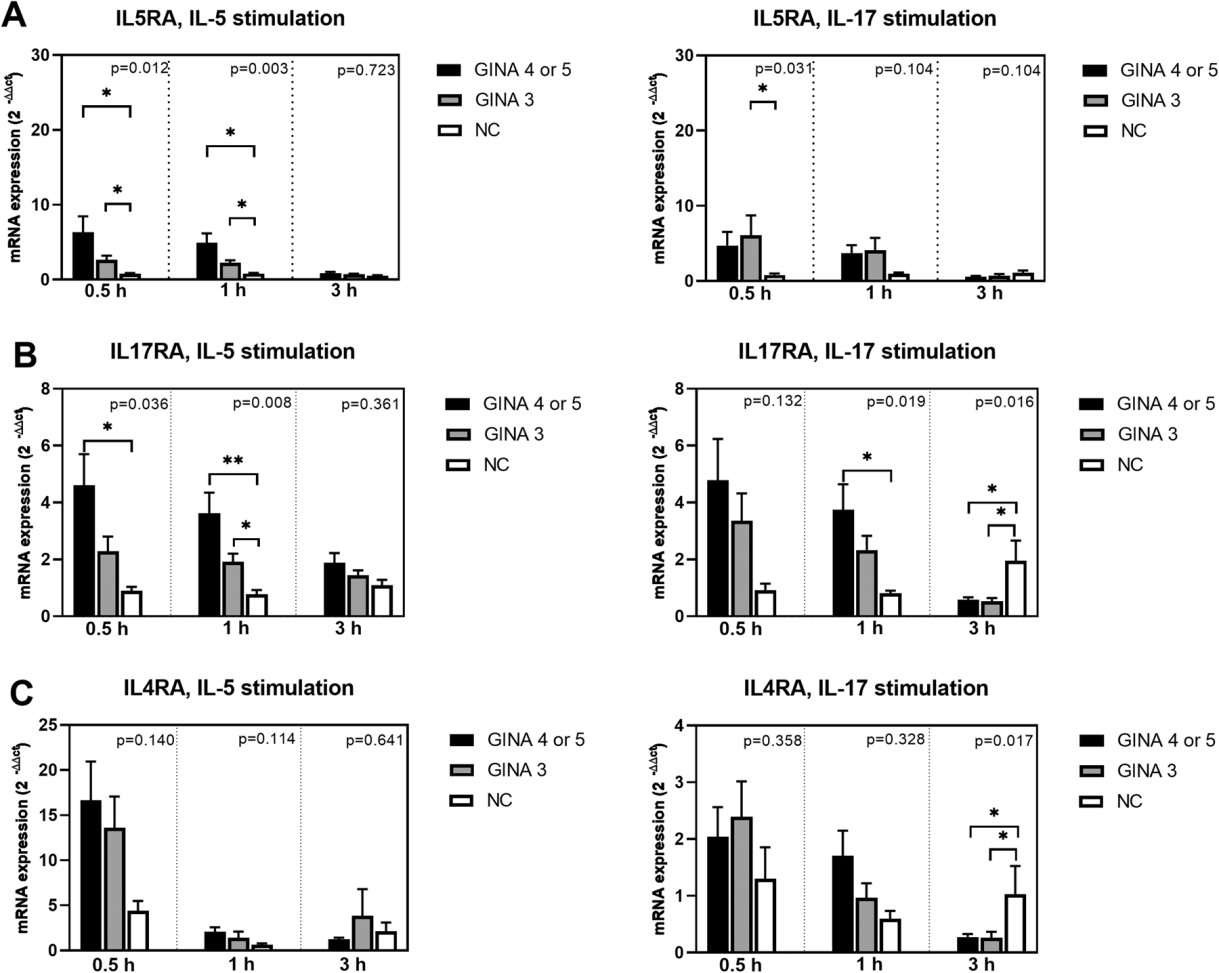

**Figure:**
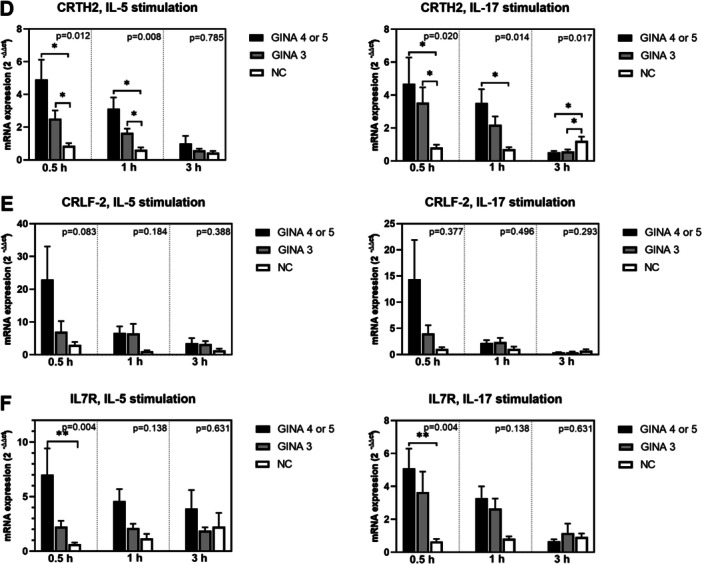

**Figure 2:**
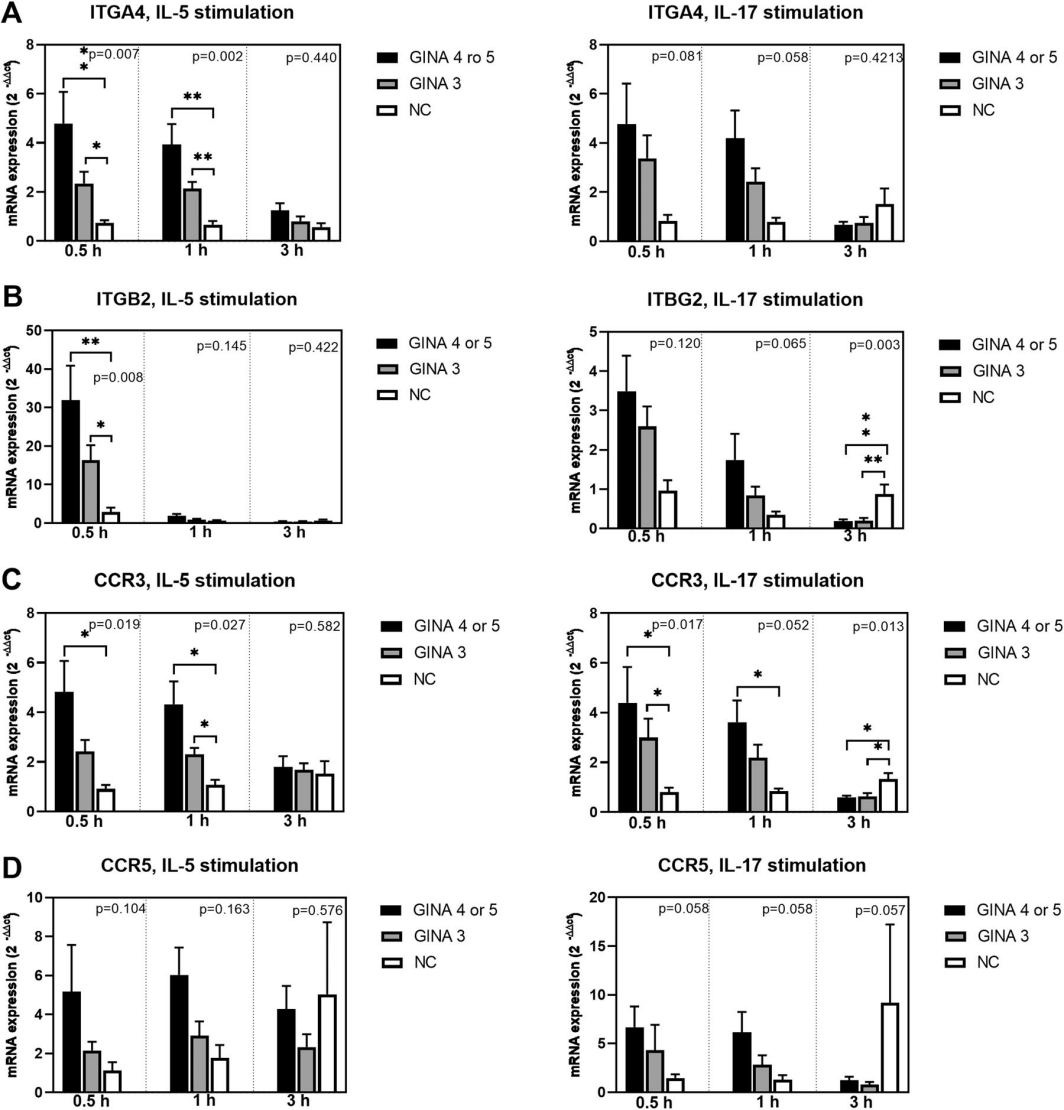
Levels of mRNA expression in eosinophils activated using interleukin‐5 or interleukin‐17 for 0.5, 1, or 3 h. (A)ITGA4. (B) ITGB2. (C) CCR3. (D) CCR5. Black bars represent patients with GINA 4 or 5, gray bars represent patients with GINA 3, and unshaded bars represent normal individuals. Post hoc analysis *p < 0.05 and **p < 0.01. CCR3, C‐C chemokine receptor 3; CCR5, C‐C chemokine receptor 5; GINA, global initiative for asthma; ITGA4, integrin‐α4; ITGB2, integrin‐β2.

Figure 2 depicts the expression levels of adhesion molecules and chemokine receptors in activated eosinophils. The expression levels of ITGA4 (Figure 2A) and ITGB2 (Figure 2B) in IL‐5‐activated eosinophils and that of CCR3 (Figure 2C) in IL‐5‐ or IL‐17‐activated eosinophils were significantly higher for patients with asthma than for normal individuals. Notably, the level of mRNA expression was higher for patients with GINA 4 or 5 than for those with GINA 3.

Figure 3 depicts the expression levels of cell function markers in activated eosinophils. The expression levels of FCER1A (Figure 3A) and TLR‐9 (Figure 3F) in IL‐5‐ or IL‐17‐activated eosinophils and those of aryl hydrocarbon receptor (Figure 3C) and TLR‐7 (Figure 3E) in IL‐5‐activated eosinophils were significantly higher for patients with asthma than for normal individuals. Notably, the level of mRNA expression was higher for patients with GINA 4 or 5 than for those with GINA 3. The expression level of TLR‐3 (Figure 3D) in eosinophils treated with IL‐5 for 1 h was significantly higher for patients with GINA 4 or 5 than for normal individuals. Furthermore, the expression level of TGFB1 (Figure 3B) in IL‐5‐activated eosinophils was significantly higher for patients with GINA 4 or 5 than for those with GINA 3 and normal individuals.

Figure 3Levels of mRNA expression in eosinophils activated using interleukin‐5 or interleukin‐17 for 0.5, 1, or 3 h. (A) FCER1A. (B) TGFB1. (C) AHR. (D) TLR3. (E) TLR7. (F) TLR9. Black bars represent patients with GINA 4 or 5, gray bars represent patients with GINA 3, and unshaded bars represent normal individuals. Post hoc analysis *p < 0.05 and **p < 0.01. AHR, aryl hydrocarbon receptor; FCER1A, Fcε receptor type I; GINA, Global Initiative for Asthma; TGFB1, transforming growth factor β1; TLR, toll‐like receptor.

**Figure:**
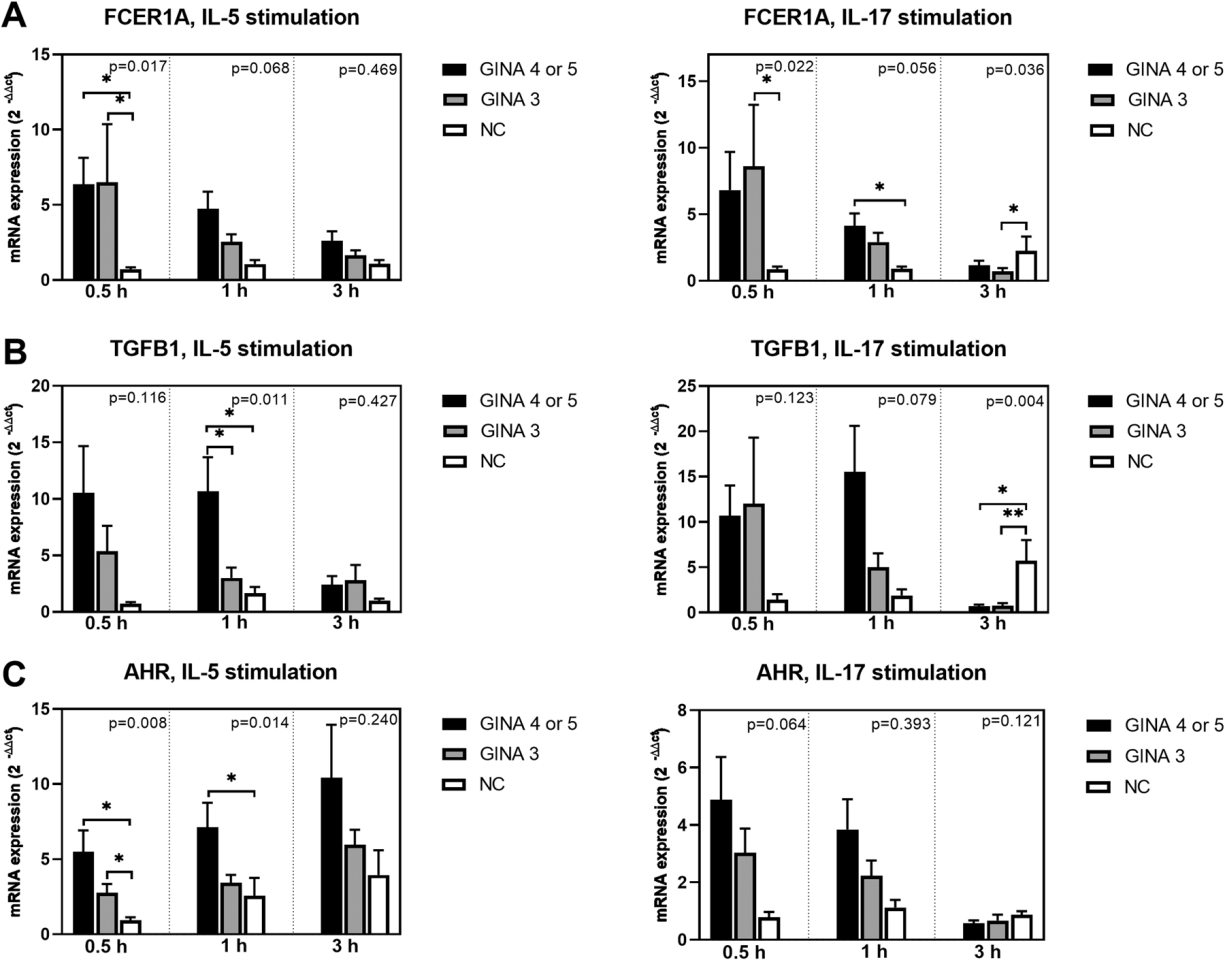

**Figure:**
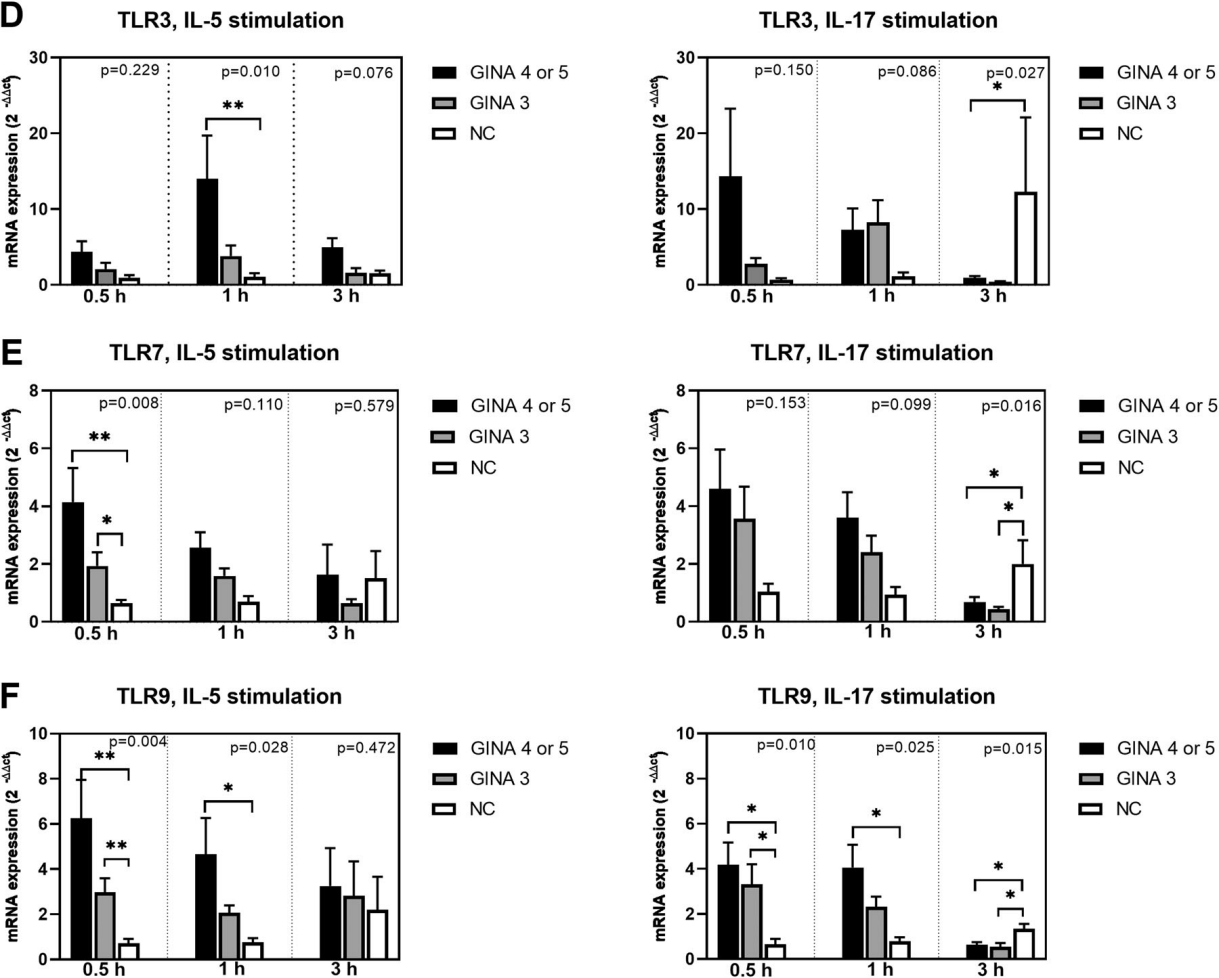

After 3‐h treatment with IL‐5 or IL‐17, the levels of mRNA expression in eosinophils derived from patients with asthma started exhibiting a declining trend. The expression levels of IL17RA, IL4RA, integrin β2, CCR3, FCER1A, TGFB1, TLR‐3, TLR‐7, and TLR‐9 (Figure 1B,C; Figure 2B,C; Figure 3A,B,D–F) in eosinophils treated with IL‐17 for 3 h were higher for normal individuals than for patients with asthma. This finding indicates that the response to IL‐17 activation is slower in normal individuals than in patients with asthma.

We also checked the mRNA expression of eosinophils treated with 30 min PBS, by the formula 2^−∆Ct^, as the baseline mRNA expression in unstimulated eosinophils Supporting Information S1: Figure S1). By this method, we did not find significant difference of genes expression of unstimulated eosinophils between groups.

### 3.3 Correlation Between Transcriptional Activity in Activated Eosinophils and Lung Function Decline in Patients With GINA 4 or 5

We first checked the baseline differences in eosinophil activation of asthma patients with low or high exposures to show if eosinophil activation increased with pollution burden. In GINA 4 or 5, eosinophils of patients with high PM_10_ exposure expressed higher baseline ITGB2, CCR5 and TLR9 than that of patients with lower PM_10_ exposure (Supporting Information S1: Figure S2). In GINA 3, there was lower baseline IL4RA and higher CRLF2 expression in eosinophils of patients with high PM_10_ exposure compared to that of patients with low PM_10_ exposure (Supporting Information S1: Figure S3). No different expression of all the other markers were found by exposure level of PM_2.5_, NO_2_ or O_3_ in patients (data not shown).

We then analyzed the mRNA expression of eosinophils after stimulation of IL‐5 or IL‐17 (0.5 h) by high or low pollutant exposure. In GINA step 4 or 5, there were higher expression of many genes after IL‐5 or IL‐17 stimulation in patients with low PM_10_ or PM_2.5_ exposure compared with that of patients with high PM_10_ or PM_2.5_ exposure (Supporting Information S1: Figure S5–S8). In GINA 3, there was no difference in mRNA expression in IL‐5 or IL‐17 stimulated eosinophils in patients with high or low PM_10_ exposure (Supporting Information S1: Figures S13 and S14). The analysis for PM_2.5_ exposure could not be done because only one GINA 3 patient belong to low PM_2.5_ exposure. There is no difference in mRNA expression of IL‐5 or IL‐17 stimulated eosinophils between high or low NO_2_ or O_3_ exposure in both groups (Supporting Information S1: Figures S15–S18).

The correlation between the level of mRNA expression in activated eosinophils and the lung function of patients with asthma was analyzed to explore the clinical implications. The level of mRNA expression peaked after 0.5‐or 1‐h treatment; therefore, these time points were considered in the correlation analysis. Table 2 presents a list of genes whose expression was significantly correlated with lung function decline. For patients with GINA 4 or 5, the expression levels of TLR‐7, TLR‐9, ITGB2, CCR3, CCR5, IL17RA, IL4RA and CRLF2 in IL‐17‐activated eosinophils and that of TLR‐3 in IL‐5‐actiavted eosinophils were correlated with lung function decline (negative correlation coefficient values). For patients with GINA 3, the expression of only CRLF2 in IL‐17‐activated eosinophils was correlated with lung function decline.

**Table 2:** Negative correlation between mRNA expression in activated eosinophils and lung function in patients receiving GINA 4 or 5 treatment and those receiving GINA 3 treatment.

**FEV1 (L) of patients with GINA 4–5**					
**Gene**	**TLR7**	**TLR9**			
Stimulation	IL‐17 (1 h)	IL‐17 (1 h)			
Spearman's *ρ*	−0.46	−0.51			
*p* Value	0.046	0.027			
**Post BD FEV1 (L) of patients with GINA 4–5**					
**Gene**	**ITGB2**	**ITGB2**	**CCR3**	**TLR7**	**TLR9**
Stimulation	IL‐17 (30 min)	IL‐17 (1 h)	IL‐17 (30 min)	IL‐17 (1 h)	IL‐17 (1 h)
Spearman's *ρ*	−0.50	−0.50	−0.48	−0.52	−0.50
*p* Value	0.031	0.048	0.044	0.021	0.029
**Gene**	**IL17RA**	**IL4RA**	**CRLF2**	**CCR5**	
Stimulation	IL‐17 (30 min)	IL‐17 (30 min)	IL‐17 (30 min)	IL‐17 (30 min)	
Spearman's *ρ*	−0.34	−0.49	−0.47	−0.48	
*p* Value	0.031	0.035	0.041	0.037	
**Change of FEV1 (%) of patients with GINA 4–5**					
**Gene**	**TLR3**				
Stimulation	IL‐5 (30 min)				
Spearman's *ρ*	−0.49				
*p* Value	0.032				
**FEV1/FVC (%) of patients with GINA 3**					
**Gene**	**CRLF2**				
Stimulation	IL‐17 (30 min)				
Spearman's *ρ*	−0.65				
*p* Value	0.026				

Abbreviations: BD, bronchodilator; CCR, C‐C motif chemokine receptor; CRLF2, cytokine receptor‐like factor 2; FEV1, forced expiratory volume in 1 s; FVC, forced vital capacity; GINA, Global Initiative for Asthma; IL17RA, interleukin‐17 receptor A; TLR, toll‐like receptor.

### 3.4 Effects of Air Pollutant Exposure on Transcriptional Activity in Activated Eosinophils From Patients With Gina 4 or 5 and Those With GINA 3 Treatment

**Figure 4:**
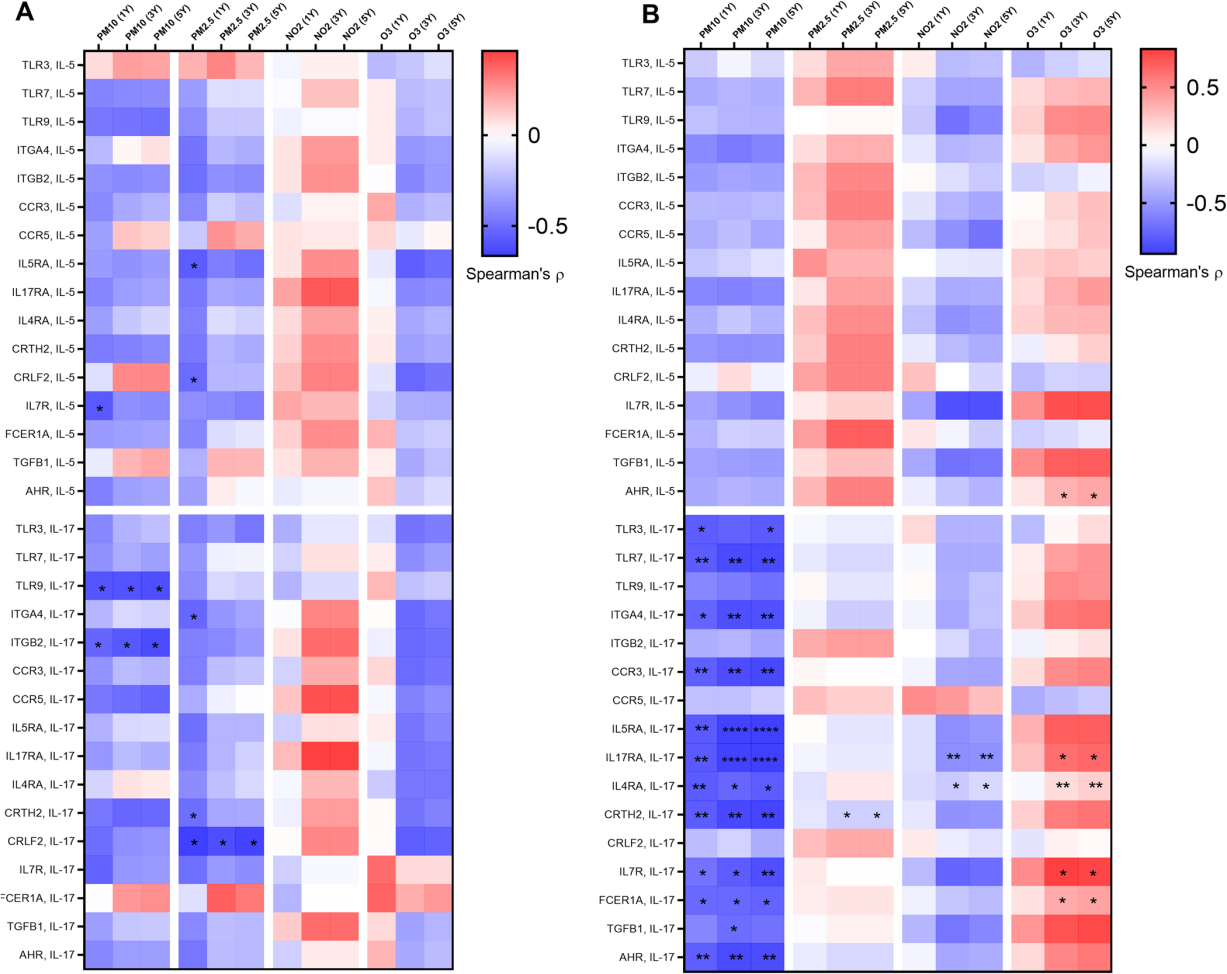
Heatmaps depicting the Spearman correlations between the expression levels of target genes in eosinophils activated using interleukin‐5 or interleukin‐17 for 0.5 h and air pollutant exposure in (A) patients with GINA 4 or 5 and (B) those with GINA 3. Red and blue indicate positive and negative correlations, respectively. The thick black frame indicates that the significance of correlations. *p < 0.05, **p < 0.01, ***p < 0.001, and ****p < 0.0001. GINA, Global Initiative for Asthma.

Heatmaps depicting the Spearman correlations between the average levels of air pollutant exposure and the expression levels of targets genes in activated eosinophils from patients with asthma are presented in Figure 4A,B. A higher correlation coefficient value indicates a stronger correlation. In general, PM_10_ exposure was negatively correlated with the expression of certain genes in eosinophils derived from the patients. For the other three air pollutants, the correlations exhibited opposite trends for the two patient groups. A positive correlation trend was noted between 3‐ and 5‐year average NO_2_ exposure levels and certain mRNA levels in patients with GINA 4 or 5. Furthermore, higher levels of PM_2.5_ and O_3_ exposure were correlated with higher levels of mRNA expression in patients with GINA 3.

**Figure 5:**
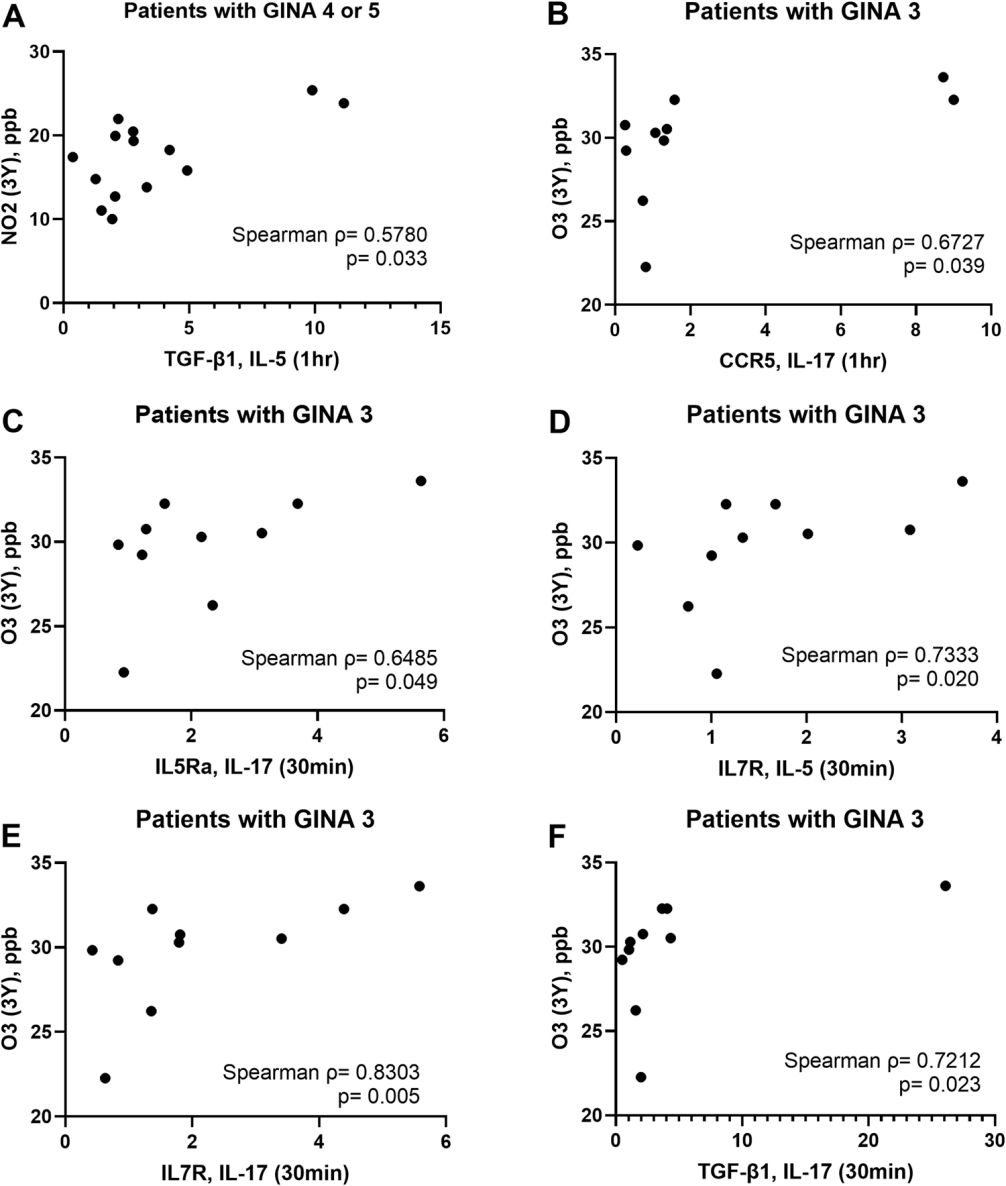
Correlations between the levels of air pollutant exposure and the expression levels of certain genes in activated eosinophils. (A) NO_2_ exposure versus TGFB1 expression in IL‐5‐activated eosinophils from patients with GINA 4 or 5. (B) O_3_ exposure versus CCR5 expression in IL‐17‐activated eosinophils from patients with GINA 3. (C) O_3_ exposure versus IL5RA expression in IL‐17‐activated eosinophils from patients with GINA 3. (D) O_3_ exposure versus IL7R expression in IL‐5‐activated eosinophils from patients with GINA 3. (E) O_3_ versus IL7R expression in IL‐17‐activated eosinophils from patients with GINA 3. (F) O_3_ exposure versus TGFB1 expression in IL‐17‐activated eosinophils from patients with GINA 3. CCR5, C‐C chemokine receptor 5; GINA, global initiative for asthma; IL‐17, interleukin‐17; IL‐5, interleukin‐5; IL5RA, interleukin‐5 receptor α; IL7R, interleukin‐7 receptor; TGFB1, transforming growth factor β1.

Figure 5 presents some examples of positive correlations between air pollutant exposure and transcriptional activity in activated eosinophils.

## 4 Discussion

In this study, the expression levels of activation‐related genes in eosinophils derived from patients with asthma were higher than those in eosinophils derived from normal individuals. Moreover, the exposure response was faster for the patients than for the controls. The expression levels of TGFB1, IL7R, and TLR‐3 in activated eosinophils were significantly higher for patients with GINA 4 or 5 than for those with GINA 3 or normal individuals. Furthermore, the expression levels of certain genes in activated eosinophils, particularly IL‐17‐activated eosinophils, were correlated with lung function decline in patients with GINA 4 or 5. The correlations between exposure to various air pollutants and transcriptional activity in eosinophils from patients with asthma exhibited different trends. Specifically, exposure to the gaseous pollutants NO_2_ and O_3_ was correlated with elevated transcriptional activity in the patients’ eosinophils. To the best of our knowledge, this study is the first to report the effects of environmental exposure on transcriptional activity in eosinophils derived from patients at various steps of GINA treatment.

The priming of blood eosinophils derived from patients managed with medium to high doses of ICSs resulted in elevated transcriptional activity compared with the priming of eosinophils from patients managed with low doses of ICSs. This finding is rare in the literature. Few studies have assessed the status of blood eosinophils in patients receiving different levels of GINA treatment. Bacci et al. reported that ICS treatment reduced the serum levels of eosinophil cationic protein in patients with asthma [23]. Grutters et al. demonstrated that ICSs inhibited the in vivo priming of blood eosinophils through cytotoxic mechanisms [24]. In the present study, the expression level of TGFB1 in IL‐5‐activated eosinophils was significantly higher for patients with GINA 4 or 5 than for those with GINA 3 and normal individuals. In addition, the expression levels of IL7R and TLR3 in activated eosinophils were higher for patients with GINA 4 or 5 than for normal individuals. TGF‐β1 is a major eosinophilic factor that is implicated in airway remodeling in patients with asthma [25]. Wong et al. revealed that TSLP significantly reduced the apoptosis of eosinophils and upregulated the expression of adhesion molecules [26]. Eosinophils are known to express TLR‐3; treatment with polyinosinic acid/polycytidylic acid can extend the survival of these cells and upregulated the expression of adhesion molecules [27]. Our findings provide novel molecular evidence for the asthma control level of GINA treatment. Further studies are required to identify the roles of TGF‐β1, IL‐7R, and TLR‐3 in asthma management.

In this study, IL‐17 induced the expression of various surface markers and TLRs in eosinophils; the higher expression levels were correlated with lower lung function parameters, particularly FEV1, in patients with GINA 4 or 5, but not in patients with GINA 3 (Table 2). This finding highlights the importance of IL‐17 in eosinophil‐mediated lung function decline. Al‐Muhsen et al. reported Th17 cytokines activate the expression TGF‐β1 in eosinophils derived from patients with asthma [28]. Notably, in our study, unlike in IL‐5‐activated eosinophils, the expression level of TGFB1 in IL‐17‐activated eosinophils was numerically, but not significantly, higher for patients with GINA 4 or 5 than for the other two groups (Figure 3B). The lack of statistical significance may be attributable to the small number of patients in our study. In a study conducted by Irvin et al., a dual‐positive Th2/Th17 subgroup of patients with asthma exhibited increased blood eosinophil counts and lung function decline; in addition, an elevated level of glucocorticoid resistance was observed in Th2/Th17 cells in vitro [29]. Our findings complement those of Irvin et al. TLR‐7, TLR‐9, and TLR‐3 have been implicated in the innate recognition of viral single‐stranded RNA, CpG‐containing DNA, and double‐stranded RNA, respectively. Activation with TLR‐7, TLR‐9, and TLR‐3 extended the survival of eosinophils and upregulated the expression of adhesion molecules in these cells [27, 30]. Future studies are warranted to investigate the role of eosinophils in airway remodeling during a viral infection–induced innate immune response. In our study, the expression of only CRLF2 in IL‐17‐activated eosinophils was correlated with reduced values of FEV1/forced vital capacity in patients with GINA 3. In a study conducted among patients with asthma, persistent airflow limitation (PAL) was detected in 59 (28.8%) of 205 patients with GINA 3 [31]. PAL is associated with advanced age, prolonged asthma duration, and elevated blood eosinophil counts. Our findings indicated the CRLF2, the receptor of TSLP, may involve in the pathogenesis of PAL in patients with GINA 3.

We identified distinct trends of correlations between exposure to various air pollutants and transcriptional activity in eosinophils derived from patients with asthma. The underling mechanisms remain to be elucidated. We found the baseline level of some genes express higher in patients with high PM_10_ exposure. However, these baseline difference cannot explain what we found in the correlation analysis (Figure 4). A significant and negative correlation was observed between PM_10_ exposure and transcriptional activity in activated eosinophils derived from patients with asthma; Lower expression of certain genes in activated eosinophils were also found in GINA 4 or 5 patients with high PM_10_ exposure. One possible explanation is the PM_10_ and PM_2.5_ exposure induces pro inflammatory state in the airway and circulation respectively, which may affect eosinophil priming. The correlation patterns for the other three pollutants varied across patients receiving different levels of GINA treatment. Mostly positive correlations in the case of PM_2.5_ in GINA3 asthma. This implies a more deleterious effect of PM_2.5_ which can freely penetrate to the blood, less visible in more severe asthma where cell priming is already very high. Notably, NO_2_ exposure was found to be correlated with upregulated TGFB1 expression in IL‐5‐activated eosinophils from patients with GINA 4 or 5. Furthermore, O_3_ exposure was found to be correlated with upregulated CCR5, IL5RA, IL7R, and TGFB1 expression in IL‐17‐activated eosinophils and upregulated IL‐7R expression in IL‐5‐activated eosinophils from patients with GINA 3. An epidemiology study revealed that exposure to NO_2_ and O_3_ is associated with wheezing and lung function decline in patients with asthma [32, 33]. NO_2_ is formed through chemical reactions between nitrogen species and reactive oxygen. Fossil fuel combustion is a key source of nitrogen and volatile compounds. O_3_ is the byproduct of a chemical reaction between volatile compounds and NO_2_. NO_2_ and O_3_ are known for their oxidative potential and low solubility, which facilitate their dispersal into the distal airway and dissolution into the alveolar lining fluid [18]. In a study conducted by Barck et al., exposure to ambient NO_2_ increased airway eosinophilia in patients with asthma who inhaled an allergen; the proportion of eosinophils was evaluated through bronchial wash and bronchoalveolar lavage [34]. Scannell et al. reported that O_3_ exposure induces neutrophilic airway inflammation in patients with asthma [35]. Our findings build upon those of the aforementioned studies by unraveling the effects of chronic exposure to gaseous pollutants on the activation of blood eosinophils in patients at various steps of GINA treatment.

This study has some limitations. First, the relatively small sample size and the cross‐sectional design of this study precludes the generalization of our findings across all populations of patients with asthma. Second, data regarding certain confounders, such as the participants’ occupational exposure, indoor pollution, and movement history, were unavailable. Thus, further studies are needed to validate our findings. Finally, a functional evaluation of transcriptional activity in eosinophils was beyond the scope of the study.

In conclusion, the expression levels of TGFB1, IL7R and TLR3 in activated eosinophils may be higher in patients with GINA 4 or 5 than in those with GINA 3 and normal individuals. IL‐17 appears to upregulate the expression of numerous surface markers and TLRs in the eosinophils of patients with GINA 4 or 5 and that of CRLF2 in the eosinophils of patients with GINA 3. This upregulation is correlated with lung function decline. Chronic environmental exposure to gaseous pollutants, such as NO_2_ and O_3_, may upregulate the expression of certain genes, such as TGFB1, in the eosinophils of patients with asthma. Our study provides new insights into eosinophil activation and lung function decline in patients with asthma who have been exposed to air pollution. Such insights underscore the need for comprehensive strategies that integrate environmental considerations into the management of asthma.

## Author Contributions

**Ting‐Yu Lin:** conceptualization, data curation, formal analysis, funding acquisition, investigation, methodology, project administration, resources, validation, visualization, writing–original draft. **Po‐Jui Chang:** data curation, investigation, methodology, project administration, resources. **Chun‐Yu Lo:** data curation, investigation, methodology, resources. **Hsiao‐Chi Chuang:** formal analysis, methodology, writing–review and editing. **Chung‐Shu Lee:** investigation, methodology, resources. **Chih‐Hao Chang:** investigation, methodology, resources. **Chih‐Teng Yu:** investigation, methodology, resources. **Meng‐Heng Hsieh:** investigation, methodology, resources. **Chien‐Ying Liu:** investigation, methodology, resources. **Chih‐Hsi Scott Kuo:** investigation; methodology; resources. **Shu‐Min Lin:** data curation, formal analysis, resources, supervision, writing–review and editing.

## Ethics Statement

All participants provided written informed consent. This study was approved by the Institutional Review Board of Chang Gung Medical Foundation (approval number: 201900943A3; approval date: October 1, 2019). All experiments were performed in accordance with the relevant guidelines and the ethical principles of the Helsinki Declaration.

## Conflicts of Interest

The authors declare no conflicts of interest.

## Supporting information

Supporting information.

## Data Availability

The data sets used and/or analyzed in this study are available from the corresponding author upon reasonable request.
