# Supplementary material for: Correlation Between mRNA Expression of Activated Eosinophils and Air Pollutant Exposure in Patients With Asthma
Source: Immun Inflamm Dis. 2024 Nov 22;12(11):e70065. doi: 10.1002/iid3.70065 (PMC11582923; doi:10.1002/iid3.70065)
Supplement: Supplementary file 1 — Supporting information. [file IID3-12-e70065-s001.docx]

**Table S1.** Primers used in quantitative reverse transcription polymerase chain reaction

| **Primer name** | **Primer Sequence** | **Orientation** |
| --- | --- | --- |
| hTLR7_qF | TCCTTGGGGCTAGATGGTTTC | fwd |
| hTLR7_qR | TCCACGATCACATGGTTCTTTG | rev |
| hTLR3_qF | GCCATGAAGTTGCTGACTGC | fwd |
| hTLR3_qR | TGGCGGCTGGTAATCTTCTG | rev |
| hTLR9_qF | CTGCCACATGACCATCGAG | fwd |
| hTLR9_qR | GGACAGGGATATGAGGGATTTGG | rev |
| hAHR_qF | CAAATCCTTCCAAGCGGCATA | fwd |
| hAHR_qR | CGCTGAGCCTAAGAACTGAAAG | rev |
| h Integrin α4_qF | TGCAAGGTTTTGTGCCCATGAA | fwd |
| h Integrin α4_qR | TCAAGTTGTACCACGCCAGAGT | rev |
| h Integrin β2_qF | AAGTGACGCTTTACCTGCGAC | fwd |
| h Integrin β2_qR | AAGCATGGAGTAGGAGAGGTC | rev |
| hCCR3_qF | TGGCATGTGTAAGCTCCTCTC | fwd |
| hCCR3_qR | CCTGTCGATTGTCAGCAGGATTA | rev |
| hCCR5_qF | TTACTGTCCCCTTCTGGGCT | fwd |
| hCCR5_qR | AAGCAAACACAGCATGGACG | rev |
| hIL5Rα_qF | TTTGCCCTTCACGCCATTGAT | fwd |
| hIL5Rα_qR | TGGATAGAGAGACGAGTTCCTTC | rev |
| hIL17RA_qF | GACACTCCGCGACTGTTTC | fwd |
| hIL17RA_qR | GCCCGTGATGAACCAGTACAC | rev |
| hIL4Rα_qF | CGTGGTCAGTGCGGATAACTA | fwd |
| hIL4Rα_qR | TGGTGTGAACTGTCAGGTTTC | rev |
| hCRTH2_qF | GACTTGGGGTCAGAGACTGC | fwd |
| hCRTH2_qR | CTTCAGTGACCCTGGCCTTT | rev |
| hCRLF2_qF | ACTCCTGTTTCAGGCATGGG | fwd |
| hCRLF2_qR | AGTCAGGTTGGTCCTGGAGT | rev |
| hIL7R_qF | CTCCAACCGGCAGCAATGTAT | fwd |
| hIL7R_qR | AGATGACCAACAGAGCGACAG | rev |
| hTGF-B_qF | GGCCAGATCCTGTCCAAGC | fwd |
| hTGF-B_qR | GTGGGTTTCCACCATTAGCAC | rev |
| hFcRIa_qF | TAAAAGCTCCGCGTGAGAAGT | fwd |
| hFcRIa_qR | AGGATGTGGGTTCAGAAGTCT | rev |
| hBeta-actin_qF | GATCATTGCTCCTCCTGAGC | fwd |
| HBeta-actin_qR | CACCTTCACCGTTCCAGTTT | rev |

Figure S1. Levels of mRNA expression levels in unstimulated eosinophils (30min PBS treatment) of asthma patients with GINA 4 or 5, with GINA 3, and normal control.

Abbreviations: IL5A, interleukin-5 receptor α; IL17RA, interleukin-17 receptor α;

IL4RA, interleukin-4 receptor α; CRTH2, prostaglandin D2 receptor 2; CRLF2, cytokine receptor-like factor 2; IL7R, interleukin-7 receptor, CCR3, C-C chemokine receptor 3; CCR5, C-C chemokine receptor 5, FCER1A, Fcε receptor type I; TGFB1, transforming growth factor β1; AHR, aryl hydrocarbon receptor; TLR, Toll-like receptor; GINA, Global Initiative for Asthma.

Figure S2. Comparison of mRNA expression levels in unstimulated eosinophils (30min PBS treatment) of patients with GINA 4 or 5 and high PM_10_ exposure versus low PM_10_ exposure.

Abbreviations: IL5A, interleukin-5 receptor α; IL17RA, interleukin-17 receptor α;

IL4RA, interleukin-4 receptor α; CRTH2, prostaglandin D2 receptor 2; CRLF2, cytokine receptor-like factor 2; IL7R, interleukin-7 receptor, CCR3, C-C chemokine receptor 3; CCR5, C-C chemokine receptor 5, FCER1A, Fcε receptor type I; TGFB1, transforming growth factor β1; AHR, aryl hydrocarbon receptor; TLR, Toll-like receptor; GINA, Global Initiative for Asthma.

Figure S3. Comparison of mRNA expression levels in unstimulated eosinophils (30min PBS treatment) of patients with GINA 3 and high PM_10_ exposure versus low PM_10_ exposure.

Abbreviations: IL5A, interleukin-5 receptor α; IL17RA, interleukin-17 receptor α;

IL4RA, interleukin-4 receptor α; CRTH2, prostaglandin D2 receptor 2; CRLF2, cytokine receptor-like factor 2; IL7R, interleukin-7 receptor, CCR3, C-C chemokine receptor 3; CCR5, C-C chemokine receptor 5, FCER1A, Fcε receptor type I; TGFB1, transforming growth factor β1; AHR, aryl hydrocarbon receptor; TLR, Toll-like receptor;; GINA, Global Initiative for Asthma.

Figure S4. Heatmaps depicting the Spearman correlations between the expression levels of target genes in eosinophils activated using interleukin-5 or interleukin-17 for 1 h and air pollutant exposure in (A) patients with GINA 4 or 5 and (B) those with GINA 3. Red and blue indicate positive and negative correlations, respectively. The thick black frame indicates that the significance of correlations. *p < .05, **p < .01, ***p < .001, and ****p < .0001.

Abbreviation: GINA, Global Initiative for Asthma.

Figure S5. Comparison of mRNA expression levels in 0.5 h IL-5 stimulated eosinophils of patients with GINA 4 or 5 by high PM_10_ exposure versus low PM_10_ exposure.

Figure S6. Comparison of mRNA expression levels in 0.5 h IL-17 stimulated eosinophils of patients with GINA 4 or 5 by high PM_10_ exposure versus low PM_10_ exposure.

Figure S7. Comparison of mRNA expression levels in 0.5 h IL-5 stimulated eosinophils of patients with GINA 4 or 5 by high PM_2.5_ exposure versus low PM_2.5_ exposure.

Figure S8. Comparison of mRNA expression levels in 0.5 h IL-17 stimulated eosinophils of patients with GINA 4 or 5 by high PM_2.5_ exposure versus low PM_2.5_ exposure.

Figure S9. Comparison of mRNA expression levels in 0.5 h IL-5 stimulated eosinophils of patients with GINA 4 or 5 by high NO_2_ exposure versus low NO_2_ exposure.

Figure S10. Comparison of mRNA expression levels in 0.5 h IL-17 stimulated eosinophils of patients with GINA 4 or 5 by high NO_2_ exposure versus low NO_2_ exposure.

Figure S11. Comparison of mRNA expression levels in 0.5 h IL-5 stimulated eosinophils of patients with GINA 4 or 5 by high O_3_ exposure versus low O_3_ exposure.

Figure S12. Comparison of mRNA expression levels in 0.5 h IL-17 stimulated eosinophils of patients with GINA 4 or 5 by high O_3_ exposure versus low O_3_ exposure.

Figure S13. Comparison of mRNA expression levels in 0.5 h IL-5 stimulated eosinophils of patients with GINA 3 by high PM_10_ exposure versus low PM_10_ exposure.

Figure S14. Comparison of mRNA expression levels in 0.5 h IL-17 stimulated eosinophils of patients with GINA 3 by high PM_10_ exposure versus low PM_10_ exposure.

Figure S15. Comparison of mRNA expression levels in 0.5 h IL-5 stimulated eosinophils of patients with GINA 3 by high NO_2_ exposure versus low NO_2_ exposure.

Figure S16. Comparison of mRNA expression levels in 0.5 h IL-17 stimulated eosinophils of patients with GINA 3 by high NO_2_ exposure versus low NO_2_ exposure.

Figure S17. Comparison of mRNA expression levels in 0.5 h IL-5 stimulated eosinophils of patients with GINA 3 by high O_3_ exposure versus low O_3_ exposure.

Figure S18. Comparison of mRNA expression levels in 0.5 h IL-17 stimulated eosinophils of patients with GINA 3 by high O_3_ exposure versus low O_3_ exposure.
